# *Hypericum triquetrifolium* and *H. neurocalycinum* as Sources of Antioxidants and Multi-Target Bioactive Compounds: A Comprehensive Characterization Combining *In Vitro* Bioassays and Integrated NMR and LC-MS Characterization by Using a Multivariate Approach

**DOI:** 10.3389/fphar.2021.660735

**Published:** 2021-03-26

**Authors:** Stefano Dall’Acqua, Gunes Ak, Kouadio Ibrahime Sinan, Fevzi Elbasan, Irene Ferrarese, Stefania Sut, Evren Yıldıztugay, Gregorio Peron, Elisabetta Schievano, Marie Carene Nancy Picot-Allain, Mohamad Fawzi Mahomoodally, Gokhan Zengin

**Affiliations:** ^1^ Department of Pharmaceutical and Pharmacological Sciences, University of Padova, Padova, Italy; ^2^ Department of Biology, Science Faculty, Selcuk University, Konya, Turkey; ^3^ Department of Biotechnology, Science Faculty, Selcuk University, Konya, Turkey; ^4^ Department of Chemical Sciences, University of Padova, Padova, Italy; ^5^ Department of Health Sciences, Faculty of Medicine and Health Sciences, University of Mauritius, Mauritius, Mauritius

**Keywords:** LC-MS, NMR, AChE, multivariate analysis, BChE, antioxidant-phytochemical studies, tyrosinase

## Abstract

*Hypericum triquetrifolium* and *H. neurocalycinum* were evaluated for their phytochemical content and *in vitro* bioactivity. NMR analyses were performed on the methanol extract of the aerial parts of *H. triquetrifolium* to establish the main classes of phytoconstituents. Then, LC-DAD-MS^n^ analyses were performed in order to compare the composition of aerial parts and roots extracts of both *Hypericum* species, obtained using either methanol or water as solvents. Results, processed using multivariate data analysis, showed a significantly higher phenolic content of methanol extracts compared to water extracts, while minor qualitative differences were observed between the two. Distinctive flavonoid and PAC patterns were observed for *H. triquetrifolium* and *H. neurocalycinum*, and specific compounds were exclusively detected in one or the other species. Specifically, the phloroglucinols 7-epiclusianone, hyperfirin and hyperforin were present only in *H. neurocalycinum,* while hyperforin was detected only in *H. triquetrifolium.* Extracts were assayed using different *in vitro* tests to evaluate their antioxidant properties and their inhibitory activity against several enzymes, showing significant antioxidant and metal chelating activities. Furthermore, inhibitory properties against acetylcholinesterase, butyrylcholinesterase and tyrosinase were observed. Multivariate approaches were used to correlate biological data with the phytochemical composition of the different extracts. The results, showing positive correlations between specific chemical constituents and the measured bioactivities, represent preliminary data that could guide future studies aimed at isolating bioactive constituents from *H. neurocalycinum* and *H. triquetrifolium* for further pharmacological evaluations.

## Introduction

*Hypericum* genus (Hypericaceae) encompasses 465 species distributed worldwide and of which nearly 100 taxa (out of which 45 are endemic) are grouped under 19 sections in Turkey (Öztürk et al., 2009; Eroglu Ozkan et al., 2018). Folk populations across the globe have been using *Hypericum* species in traditional medicine for centuries, and even nowadays the therapeutic potential of the same species is harnessed in complementary and alternative medicine. *Hypericum perforatum* L., commonly known as St. John’s Wort, is the most studied species of the *Hypericum* genus. Comprehensive reviews have been published highlighting its applications in nutraceutical, phytopharmaceutical and cosmetic products. Furthermore, numerous biological activities of *H. perforatum* L. have been studied, including antibacterial, antiviral, antidepressant and pain-relieving (Agostinis et al., 1995; Biffignandi and Bilia, 2000; Saddiqe et al., 2010; Coppock and Dziwenka, 2016; Galeotti, 2017). The increase in research awareness towards *Hypericum* species might have been nurtured by the interesting pharmacological activity of *H. perforatum* L., which has led to advanced clinical research. Although a meaningful number of scientific studies have focused on the phytochemical composition and bioactivity of numerous *Hypericum* species, some of them still require more scientific assessment and validation due to limited information.

Petroleum ether and methanol extracts of the flowering aerial parts of *H. neurocalycinum* Boiss. & Heldr., endemic to Turkey, have been previously reported as potential antimicrobial agents against methicillin resistant *S. aureus* and *S. epidermidis,* while demonstrating low toxicity against HeLa and NRK-52E cell lines (Ozkan et al., 2013; Özkan et al., 2019). The methanol extract of *H. triquetrifoium* Turra aerial parts, a species known as curled-leaved St. John's Wort and distributed in the Mediterranean basin (Volkov, 2018), has been reported to exert anti-inflammatory activity in carrageenan-induced paw edema rats and antinociceptive activity in mouse model challenged with formalin (Apaydın et al., 1999; Ozturk et al., 2002). I3-II8-biapigenin isolated from *H. triquetrifolium* aerial parts exhibited cytotoxic activity against amelanotic melanoma cell line C32 and large cell lung carcinoma cell line COR-L23, with IC_50_ of 5.73 and 37.42 mg/ml, respectively (Conforti et al., 2007).

The present study aimed at investigating and comparing the phytochemical composition and the antioxidant and enzyme inhibitory potential of two *Hypericum* species of the Turkish flora, namely *H. neurocalycinum* and *H. triquetrifolium*. NMR technique was used to preliminary investigate the class of metabolites in *H. triquetrifolium* extracts. NMR data allowed to set up an appropriate integrated liquid chromatography coupled to diode array detector and multi-step tandem mass spectrometry (LC-DAD-MS^n^) and LC coupled to quadrupole-time of flight MS (QTOF) method for the comprehensive phytochemical characterization of methanol and water extracts prepared from both aerial parts and roots of the two *Hypericum* species. Finally, the results from phytochemical screening and *in vitro* bioassays were analyzed using multivariate techniques, in order to find correlations between specific chemical constituents and the monitored bioactivities.

## Materials and Methods

### Plant Material and Preparation of Extracts

The plant material for extraction was collected in the area of Turkey during summer 2019 (*H. neurocalycinum*: Hadim village, Dedemli Valley, 3,140 m, Konya; *H. triquetrifolium*: Anamur village, the ancient city of Anemurium, 5 m, Mersin). Taxonomic identification was performed by the botanist Dr. Evren Yıldıztugay (Selcuk University, Department of Biotechnology, Konya, Turkey) and one voucher specimen for each species (voucher ID numbers: EY-3110 for *H. neurocalycinum* and EY-3072 for *H. triquetrifolium*) was deposited at the herbarium of Selcuk University. The aerial parts (flowers, leaves and stem as mix) and roots were carefully separated. Then, plant materials were dried in a shaded and well-ventilated environment. After drying (about 10 days), plant materials were powdered using a laboratory mill. Powdered plant materials were stored in a dark and cool place and they were kept away from sunlight.

In the study, maceration was preferred to obtain methanol extract. Maceration could be useful to extract thermolabile compounds and this method could be easily performed in further applications. Briefly, powdered plant samples (5 g) were stirred with 100 ml of methanol for 24 h at room temperature. Afterwards, the mixture was filtered and the solvent was evaporated by using rotary-evaporator. Infusion was selected for water extracts. Briefly, the material (5 g) was kept in boiled water (100 ml) for 15 min, then the extract was filtered and lyophilized. Obtained dry extracts were stored at 4°C (Etienne et al., 2021; Sinan et al., 2021).

### Determination of Total Phenolic and Total Flavonoid Contents

Spectrophotometric methods were used to determine total phenolic and flavonoid contents, as already reported in earlier papers. Standard equivalents (gallic acid equivalent: GAE, for phenolics; rutin equivalent: RE, for flavonoids) were used to explain the contents in the plant extracts (Slinkard and Singleton, 1977; Zengin et al., 2016).

### Phytochemical Investigations

For the preliminary NMR analyses, a sample of *H. triquetifolium* methanol extract was dissolved in methanol/water (50%) mixture (22.5 mg/ml) and the solution was loaded on a Bondelute C-18 solid phase extraction (SPE) cartridge (3 ml). Cartridge was washed with water (2 column volumes), then compounds were eluted using methanol/water (2 volumes) and methanol (2 volumes).

#### NMR Spectroscopy

NMR spectra were recorded at 600 MHz on Bruker Avance NEO spectrometer equipped with a Cryo probe Prodigy TCI 5 mmm. All experiments were performed at 298 K. COSY, TOCSY, edited-HSQC, HMBC spectra were obtained using gradient selected pulse sequences. The spectral widths were 7,000 and 25,000 Hz for the ^1^H- and ^13^C-dimensions, respectively. The number of collected complex points was 1,024 for ^1^H-dimension with a recycle delay of 1.5 s. TOCSY experiments were acquired with 16 transients, 512 increments in second dimension and a 70 ms of spin lock period. Heteronuclear spectra were acquired with 64–96 transients, and 140–200 time increments in ^13^C-dimension. HSQC experiments used a one-bond carbon-proton coupling constant of 145 Hz, HMBC experiments used a long-range carbon-proton coupling constant of 8 Hz. 2D spectra were processed (software Topspin 4.0.6, Bruker BioSpin) using zero filling to 1,024 in F1 dimension, squared sine-bell apodization in both dimensions, prior to Fourier transformations.

#### LC-DAD-MS^n^ (Ion Trap) and UPLC-QTOF Analyses

LC-DAD-MS^n^ analyses were obtained using an Agilent LC system (Series 1260) equipped with DAD, autosampler and column oven. After the chromatographic column, a “T” connection splitted the flow equally to DAD and MS. As mass spectrometer, a Varian MS 500 Ion trap equipped with Electrospray Ion Source (ESI) was used, working in negative ion mode and acquiring the data in the *m/z* range 100–2,000. Fragmentation of most intense ion species was obtained using the turbo data depending scanning (tdds^®^) function of the instrument. Parameters were as follows: spray shield, 600 V; nebulizer pressure, 25 psi; drying gas pressure, 15 psi; capillary voltage, 80 V; RF loading, 80%; needle voltage, 4,500 V. An Agilent XDB C-18 column (3.0 × 150 mm, 3.5 µm) was used as stationary phase. Solvents were: 1% formic acid in water A), acetonitrile B) and methanol C). Gradient was as follows: 0 min, 98% A and 2% B; isocratic up to 5 min; 25 min, 80% A, 10% B, and 10% C; 40 min, 60% A, 30% B, 10% C; 45 min, 20% A, 70% B, and 10% C; isocratic up to 60 min. The flow rate was 400 ml/min.

As reference compounds for quantitative analyses, chlorogenic acid, gallic acid, epicatechin, quercetin-3-glucoside, quercetin, hyperoside, rutin, hypericin and hyperforin were used, and calibration curves were built. Chlorogenic acid solutions were used for quantification of hydoxycinnamic derivatives at 330 nm, and the calibration curve was *y* = 165.6*x* − 382.1 (*R* = 0.99991). For the quantification of small phenolics, catechin and procyanidin derivatives, gallic acid and epicatechin solutions were used and analyzed at 280 nm. Calibration curves were *y* = 122.2*x* + 16.0 (*R* = 1) and *y* = 27.8*x* + 111.6 (*R* = 0.9908), respectively. Quercetin, quercetin-3-glucoside, hyperoside and rutin solutions were used for the quantification of flavonoid and flavonoid glycosides, and they were analyzed at 280 nm. Calibration curves were *y* = 80.9*x* – 74.4 (*R* = 0.9999), *y* = 39.3*x* + 227.1 (*R* = 0.9889), *y* = 89.9*x* + 417.9 (*R* = 0.9964) and *y* = 39.2*x* + 19.6 (*R* = 0.9996), respectively. Naphthodianthrone derivatives were quantified with hypericin solutions at 590 nm, and the calibration curve was *y* = 266*x* + 8,199 (R = 0.9988). Quantification of phloroglucinols was obtained using MS. Hyperforin solutions were used, and the calibration curve was *y* = 5.58e^+5^ *x* + 1.14e^+7^ (*R* = 0.9999). Solutions were prepared in the range of 100–0.1 μg/ml.

Identification of compounds was obtained comparing the MS fragmentation spectra with the literature, and MS and retention times (R.T.) with those of available standard compounds.

Accurate *m/z* values were obtained using a Waters Acquity UPLC system coupled to a Waters Xevo G2 QTOF MS detector, operating in ESI (-) mode. For chromatographic separation, an Agilent Eclipse plus C18 column (2.1 × 50 mm, 1.8 µm) was used as stationary phase, and a gradient mixture of methanol 1) and 0.1% formic acid in water 2) as mobile phase. The gradient was: 0 min, 2% A; 0.75 min, 2% A; 11 min, 100% A; 13.5 min, 100% A; 14 min, 2% A and isocratic up to 15 min. Flow rate was 0.4 ml/min. MS parameters were as follows: sampling cone voltage, 40 V; source offset, 80 V; capillary voltage, 3,500 V; nebulizer gas (N_2_) flow rate, 800 L/h; desolvation temperature, 450°C. The mass accuracy and reproducibility were maintained by infusing lockmass (leucine-enkephalin [M–H]^−^ = 554.2620 m*/z*) thorough Lockspray at a flow rate of 20 μl/min. Centroided data were collected in the *m/z* range 50–1,200, and the *m/z* values were automatically corrected during acquisition using lockmass.

### Determination of Antioxidant and Enzyme Inhibitory Effects

Different protocols were performed to explain the antioxidant properties of *Hypericum* extracts. The protocols included reducing power (CUPric Reducing Antioxidant Capacity assay: CUPRAC and Ferric Antioxidant Power assay: FRAP), metal chelating, phosphomolybenum and free radical scavenging (2,2-diphenyl-1-picryl-hydrazyl-hydrate assay: DPPH; and 2,2′-azino-bis(3-ethylbenzothiazoline-6-sulfonic acid) assay: ABTS). Experimental details were given in our previous paper (Grochowski et al., 2017). Inhibitory effects of *Hypericum* extracts were tested against different enzymes (tyrosinase, α-amylase, α-glucosidase and cholinesterase). Both antioxidant and enzyme inhibition assays were explained by standard equivalents (trolox and EDTA for antioxidant; galantamine for cholinesterase; kojic acid for tyrosinase; acarbose for amylase and glucosidase) (Grochowski et al., 2019; Stojković et al., 2020).

### Data Analysis

One-way ANOVA followed by Turkey post-hoc test was performed to assess the difference between the averages of the samples. The analysis was performed using XLSTAT software v. 2018. The *p*-value for each parameter was evaluated, and a *p* < 0.05 was considered as statistically significant. After the univariate analysis, a supervised PLS-DA analysis was carried out through the *R* package mixOmics to discriminate the two studied species. The variable importance on projection (VIP) score of each bioactivity was calculated to reveal the most discriminant, and Student’s t-test was performed to compare the species considering those discriminant bioactivities.

### Correlation Analysis

Correlation analysis between phenolic compounds identified in *Hypericum* methanol extracts and biological activities was performed using the Spearman rank correlation test. Data pre-processing included the removal of variables with more than 80% missing values, the imputation of the remaining missing values using the *K*-nearest neighbors (KNN) algorithm, and finally data normalization by means of *log* transformation and Pareto scaling. Analysis was performed using the Metaboanalyst v. 4.0 platform (Chong et al., 2019).

## Results and Discussion

### Total Bioactive Components

Supplementary Table S1 summarizes the total phenolic and flavonoid contents of methanol and water extracts of the aerial parts and roots of *H. triquetrifolium* and *H. neurocalycinum*. In most of the cases, phenolic and flavonoid contents of the methanol extracts were significantly (*p* < 0.05) higher compared to water extracts. This finding has been already reported in several studies (Boeing et al., 2014; Osmić et al., 2018). In addition, it was also noted that phenolic and flavonoid contents of the aerial parts of both *Hypericum* species were significantly higher than the roots (Supplementary Table S1). Thus, on the basis of these preliminary data, phytochemical investigations were initially performed on *H. triquetifolium* aerial parts.

### NMR Analysis

An initial screening on *H. triquetifolium* was performed using different NMR approaches. ^1^H NMR was acquired on the methanol extracts of the aerial parts of *H. triquetifolium* (ATM) after partial fractionation obtained by C-18 SPE. Extract was suspended in water and loaded in the column. After washing with water, a first methanol/water (50–50%) fraction and a 100% methanol fraction were eluted. NMR analyses of the two fractions were used to support the elucidations of the main constituent present in the extract, and to compare the data obtained in LC-DAD-MS^n^.

### NMR Analysis of Methanol/Water (50–50%) ATM Fraction

**FIGURE 1:**
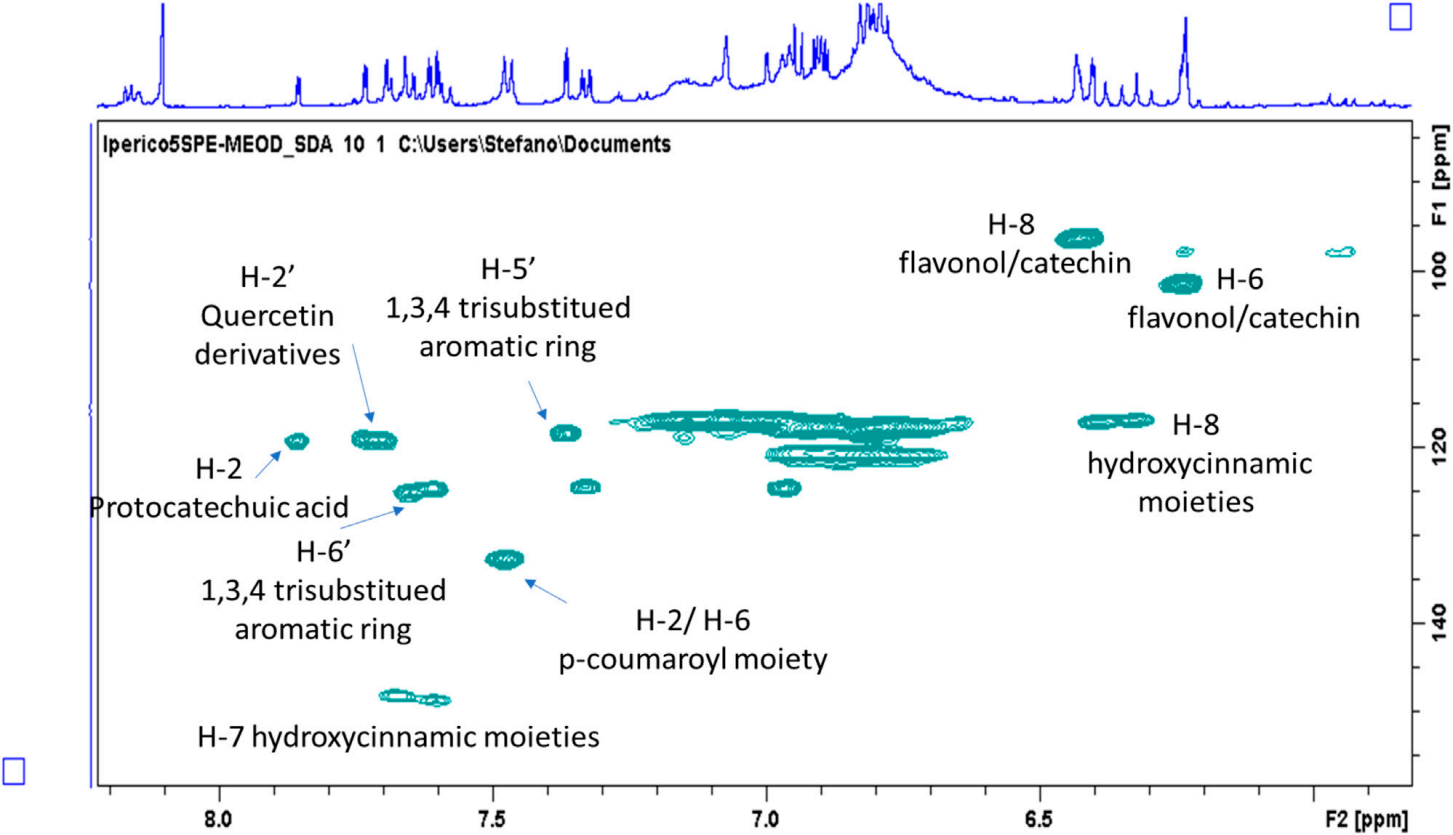
Enlargement of the HSQC-DEPT of the methanol/water (50–50%) ATM fraction with highlighted resonances assigned to principal classes of phenolic constituents.

**FIGURE 2:**
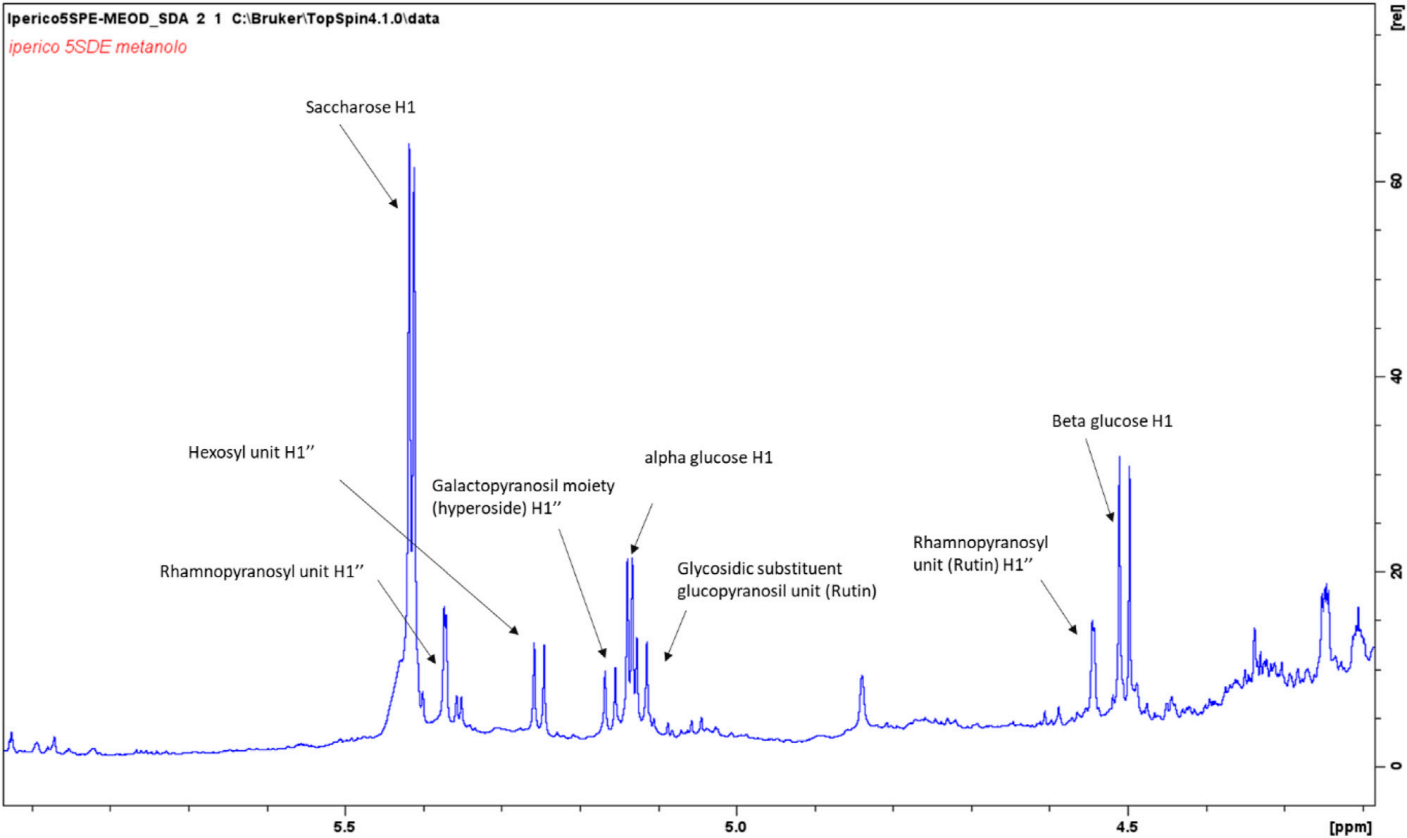
Enlargement of the ^1^H NMR spectrum of the methanol/water (50–50%) ATM fraction with highlighted signals assigned to principal anomeric sugar protons.

The aromatic part presents a large number of signals that support the presence of hydroxycinnamic acid, *p*-coumaric acid, quercetin and a protocatechuic acid derivative. Main assignments were deduced combining the data obtained from 2D-NMR experiments as well as comparing literature data and reference compounds. Figure 1 represents an enlarged portion of the edited-HSQC spectrum with the main assignments highlighted, while Supplementary Figure S1 reports the chemical structures of the identified compounds. Further signals are observed in the spectrum range δ 5.00–3.00 and most of the constituents can be ascribed to sugars as α- and β-glucose, fructose and sucrose. Signals in the spectral region δ 5.5–4.5 can be ascribed to the anomeric proton of glycosidic substituents (Figure 2). Main assignments are reported in Supplementary Table S2.

### NMR Analysis of 100% Methanol ATM Fraction

Methanol fraction eluted from SPE resulted less rich in compounds compared to the methanol/water one. Intense signals in the aliphatic part can be ascribed to fatty acids, as expected. Signals supporting the presence of phloroglucinols were detected, and main assignments are reported in Supplementary Table S3. Considering the reported phloroglucinols for different *Hypericum* specie*s* (Porzel et al., 2014), signals were assigned to hyperforin-type derivatives. In particular, signals supporting the presence of three prenyl moieties were observed, mostly due to the three sp^2^ olefinic CH that showed COSY coupling with a signal in the aliphatic part of the spectrum (δ 1.65), deriving from a quaternary methyl group. Signals suggesting the presence of a keto-isobutyl moiety were also observed. Diagnostic HMBC correlations were observed from singlet at δ_H_ 1.05 (δ_C_ 16.5) assigned to methyl group 31, with keto functions at δ_C_ 210.0, 208 as well as with quaternary carbon at δ_C_ 55.0 (C-1). Further diagnostic HMBC were observed from methyl group 14 with C-6 (δ 55.0), C-5 (δ 83.7), C-7 (δ 45.0) and C-15 (δ 37.0). Comparison with literature (Porzel et al., 2014) suggested the presence of hyperpolyphyllirin as one of the most abundant derivatives.

### LC-DAD-MS^n^ Phytochemical Analysis of the Different *Hypericum* Extracts

Once established the different classes of compounds by NMR, an integrated LC-DAD-MS^n^ (ion trap) and LC-QTOF (high resolution) approach was used to compare the phytochemical composition of roots and aerial parts extracts of the two *Hypericum* species obtained with water and methanol as solvents. Compound identification was performed by combining the data from DAD, ion trap (MS^n^) and high resolution MS and comparing the results from NMR. Identified compounds could be classified in four different classes of phytoconstituents.

#### Phloroglucinols

*Hypericum* species are known for containing different isoprenyl phloroglucinols, in many cases related to hyperforin (Tawaha et al., 2010; Pang et al., 2020). In our analysis, hyperforin was used as reference compound in order to assess main fragmentation pathways and to compare the MS behaviour of other detected phloroglucinols (Table 1).

**TABLE 1:** LC-DAD-MS^n^ and LC-QTOF data used for the identification of phloroglucinols in *Hypericum triquetrifolium* and *Hypericum neurocalycinum* methanol and water extracts of both aerial parts and roots.

R.T. (min)	[M-H]^-^	MS2 *	Theoretical *m/z*	Exp. HR *m/z* **	Δppm	Molecular Formula ([M-H]^-^)	HT-AP-MeOH	HT-AP-Water	HN-AP-MeOH	HN-AP-Water	HT-R-MeOH	HT-R-Water	HN-R-MeOH	HN-R-Water	Identification [reference]
50.4	359		359.2222	359.2225	−0.88	C22H31O4	ND	ND	ND	ND	ND	0.02 ± 0.01	ND	0.15 ± 0.01	1′3′pren45′me4′oxoPIB (Crispin et al., 2013)
54.4	331	287 (217,151) 262,207	331.1909	331.1909	0.00	C20H27O4	1.63 ± 0.03	0.04 ± 0.01	0.23 ± 0.01	ND	ND	ND	1.23 ± 0.01	0.12 ± 0.01	Geranyl phlorisobutyrophenone (Porzel et al., 2014)
54.7	497	427,357,276 233 207	497.3267	497.3276	–1.92	C31H45O5	1.12 ± 0.04	0.04 ± 0.01	ND	ND	ND	ND	1.07 ± 0.04	ND	Garsubellin E (Fukuyama et al., 1998)
56.4	501	432 (363,327,305 271)	501.3005	501.2993	2.54	C33H41O4	ND	ND	0.47 ± 0.01	0.57 ± 0.01	ND	ND	9.94 ± 0.06	1.78 ± 0.04	7-Epiclusianone (Porzel et al., 2014)
57.7	481	437 (369,245) 411,369 301 277 233	481.3318	481.3325	−1.58	C31H45O4	6.74 ± 0.06	3.36 ± 0.01	ND	ND	0.87 ± 0.02	0.33 ± 0.01	ND	ND	Hyperpolyphyllirin (Porzel et al., 2014)
58.7	467	423,398 (329,277,219) 329,287	467.3161	467.3159	0.45	C30H43O4	ND	ND	3.20 ± 0.01	3.24 ± 0.03	ND	ND	6.07 ± 0.05	6.10 ± 0.05	Hyperfirin (Porzel et al., 2014)
59.3	481	437 (369,245) 411,369 301 277 233	481.3318	481.3323	–1.14	C31H45O4	1.64 ± 0.04	0.69 ± 0.01	ND	ND	0.12 ± 0.01	ND	ND	ND	Hyperpolyphyllirin isomer (Porzel et al., 2014)
59.4	535	467,451,398 384 327 271 234	535.3787	535.3775	2.40	C35H51O4	ND	ND	0.01 ± 0.01	0.01 ± 0.01	ND	ND	0.24 ± 0.01	0.04 ± 0.01	Hyperforin (Alali et al., 2009; Porzel et al., 2014)
59.8	549	413 (369,343,327 271)	549.3944	549.3931	2.50	C36H53O4	ND	ND	0.08 ± 0.01	0.08 ± 0.01	ND	ND	ND	1.34 ± 0.01	Adhyperforin [16]

HT-AP-MeOH: *H. triquetrifolium* aerial parts, methanol extract; HT-AP-Water: *H. triquetrifolium* aerial parts, water extract; HT-R-MeOH: *H. triquetrifolium* root, methanol extract; HT-R-Water: *H. triquetrifolium* root, water extract; HN-AP-MeOH: *H. neurocalycinum* aerial parts, methanol extract; HN-AP-Water: *H. neurocalycinum* aerial parts, water extract; HN-R-MeOH: *H. neurocalycinum* root, methanol extract; HN-R-Water: *H. neurocalycinum* root, water extract; ND: not detected. *: fragments in bold indicate the source of the MS3 fragments, reported in brackets; **: experimental values obtained from LC-QTOF analysis.Quantitative data of all the extracts are also reported.

Considering hyperforin (R.T. 59.4 min), the most abundant fragment in MS^2^ is ascribable to the loss of a prenyl (3-methylbut-2-en-1-yl) unit (− 69 a.m.u.), leading to the fragment ion at *m/z* 467. Further ions formed by the MS^2^ fragmentation of the *m/z* 467 ion were observed at *m/z* 315 and 313. The fragment at *m/z* 315 could be explained with the neutral loss of two prenyl units and one 4-methylpent-3-en-1-yl one (− 83 a.m.u.). The fragment at *m/z* 313 can be ascribed to the loss of one prenyl unit, the isopropenyl moiety (−71 a.m.u.) and 4-methylpent-3-en-1-yl one (−83 a.m.u.). Other significant fragments were observed: one at *m/z* 451.8, corresponding to the loss of 4-methylpent-3-en-1-yl; one at *m/z* 398, corresponding to the loss of two 3-methylbut-2-en-1-yl units (−138 a.m.u.); one at *m/z* 384, corresponding to the loss of both prenyl and isoprenyl moieties.

MS^3^ data from *m/z* 398 showed fragments at *m/z* 354 and 352, corresponding to the loss of CO_2_ (−44 a.m.u.) and HCOOH (−46 a.m.u.) from the keto-enolic moiety of hyperforin. A lower molecular weight fragment at *m/z* 259 was observed after the fragmentation of the ion at *m/z* 398, due to the loss of two 3-methylbut-2-en-1-yl units (−138 a.m.u.). Ions at *m/z* 315 and 313 were formed by the fragmentation of the species at *m/z* 384, as observed for the parent ion at *m/z* 467, together with small fragments at *m/z* 271 (−113 a.m.u.), ascribable to the loss of CO_2_ and 3-methylbut-2-en-1-yl (−69 a.m.u.).

Hyperfirin was assigned to the compound eluted at R.T. 58.7 min, presenting *m/z* 467 and similar fragmentation pattern as hyperforin, as suggested by previous publications (Porzel et al., 2014).

**FIGURE 3:**
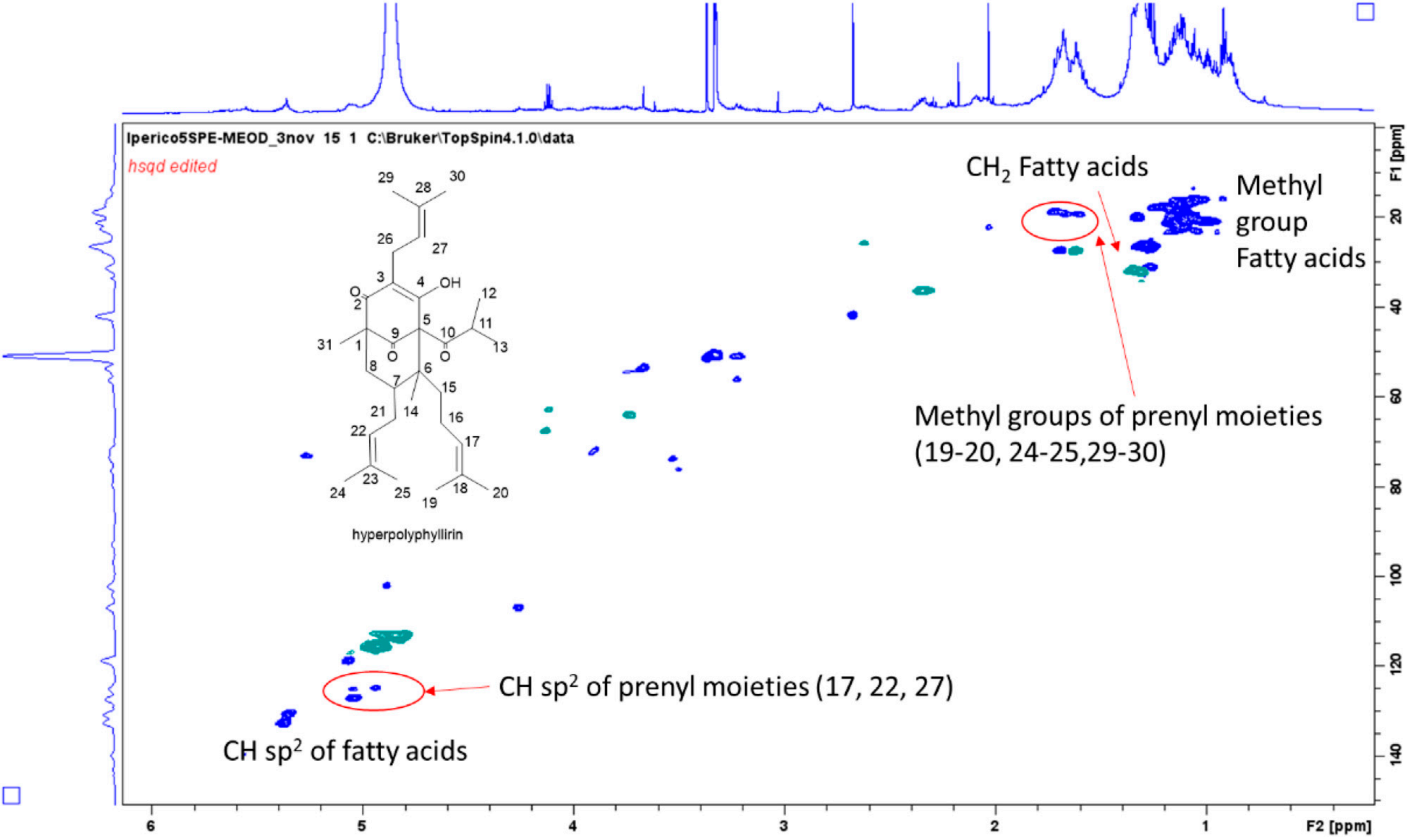
HSQC-DEPT-NMR spectrum of the methanol ATM fraction of *H. triquetifolium* aerial parts obtained by SPE. The structure of hyperpolyphyllirin and some of the assigned positions are highlighted.

Compounds at *m/z* 481 were observed at R.T. 57.7 min and 59.3 min. Both the peaks showed MS^2^ fragments related to loss of CO_2_ (*m/z* 437) and a prenyl moiety (*m/z* 411), together with other signals at *m/z* 276 (corresponding to the loss of 205 a.m.u.) and 233 (−249 a.m.u.), being this latter common to other phloroglucinol derivatives (Porzel et al., 2014). The difference with hyperforin was related to the loss of CO_2_ in MS^2^, while in the fragmentation of hyperforin this loss was observed only in MS^3^ after the loss of a prenyl unit, namely from the ion at *m/z* 467. We could suggest that these derivatives are similar to hyperforin, but with a missing prenyl unit. On the basis of the HSQC-DEPT-NMR spectrum of the methanol ATM fraction obtained by SPE (Figure 3) and previously published MS data (Porzel et al., 2014), the structure of the derivatives could be assigned to hyperpolyphyllirine. Due to the presence of the two chromatographic peaks, we could suggest that one is ascribable to hyperpolyphyllrine and one to an isomer.

Based on literature data and on the MS fragmentations reported in Table 1, tentative identification of 1′3′pren45′me4′oxoPIB, geranyl phlorisobutyrophenone, garsubellin E, 7-epiclusianone and adhyperforin were annotated. To estimate the amount of phloroglucinol derivatives in the different extracts, hyperforin was used as reference compound. As reported in Table 1, hyperforin was detected in the root and aerial parts extracts of *H. neurocalycinum*. Other derivatives detected only in this species included 7-epiclusianone, hyperfirin and adhyperforin, mostly in roots. On the other hand, hyperpolyphyllirine and its isomer were observed only in *H. triquetifolium,* mostly in the aerial parts.

#### Hydroxycinnamic Derivatives

LC-DAD-MS^n^ and LC-QTOF analyses of both *H. triquetifolium* and *H. neurocalycinum* aerial and root extracts revealed numerous peaks with UV spectra and *m/z* values corresponding to hydroxycinnamic derivatives. These were eluting in the first part of the chromatogram, i.e. between 13–28 min. Peaks at R.T. 13.2, 22.9 and 23.5 min presenting *m/z* 353 were ascribable to 1-caffeoylquinic acid, 3-caffeoylquinic acid and 5-caffeoylquinic acid, respectively, due to their fragmentation patterns (Clifford et al., 2003). Several peaks with [M-H]^-^ at *m/z* 337 were assigned to *p*-cumaroylquinic acid derivatives. LC-MS structural information are reported in Table 2. Overall, our findings are consistent with previously published data on *H. triquetifolium* and *H. neurocalycinum*, reporting caffeoyl- and p-coumaroylquinic acid conjugates among the most abundant phytoconstituents (Ozkan et al., 2013; Karakashov et al., 2015).

**TABLE 2:** LC-DAD-MS^n^ and LC-QTOF data used for the identification of phenolic constituents in *Hypericum triquetrifolium* and *Hypericum neurocalycinum* methanol and water extracts of both aerial parts and roots. Quantitative data of all the extracts are also reported.

R.T. (min)	[M-H]^-^	MS2 fragmentation *	Theoretical *m/z*	Experimental HR *m/z* **	Δppm	Molecular Formula ([M-H]^-^)	Identification	HT-AP-MeOH	HT-AP-Water	HN-AP-MeOH	HN-AP-Water	HT-R-MeOH	HT-R-Water	HN-R-MeOH	HN-R-Water
Hydroxycinnamic acids															
13.2	353	191,179,135	353.0873	353.0869	1.08	C16H17O9	1-Caffeoylquinic acid	36.12 ± 0.08	61.29 ± 0.21	17.81 ± 0.08	15.09 ± 0.05	2.16 ± 0.08	1.89 ± 0.06	3.37 ± 0.08	5.71 ± 0.03
16.5	341		341.0872	341.0872	0.00	C15H17O9	Caffeoyl hexose	ND	ND	ND	4.78 ± 0.08	0.07 ± 0.05	ND	1.42 ± 0.05	1.53 ± 0.05
17.3	337		337.0923	337.0922	0.31	C16H17O8	3-*p*-Cumaroylquinic acid	11.00 ± 0.18	22.78 ± 0.08	7.37 ± 0.09	ND	0.08 ± 0.04	0.38 ± 0.04	ND	ND
17.9	337	163,119 93	337.0923	337.0920	0.94	C16H17O8	trans-5-*p*-Cumaroylquinic acid	20.00 ± 0.08	18.36 ± 0.01	0.29 ± 0.04	1.48 ± 0.05	2.27 ± 0.09	1.54 ± 0.03	0.31 ± 0.05	0.46 ± 0.05
19.1	337		337.0923	337.0922	0.31	C16H17O8	4-*p*-Cumaroylquinic acid	1.44 ± 0.08	1.09 ± 0.01	1.92 ± 0.01	1.01 ± 0.08	ND	ND	ND	ND
20.1	337		337.0923	337.0921	0.63	C16H17O8	1-*p*-Cumaroylquinic acid	1.15 ± 0.08	34.18 ± 0.07	5.10 ± 0.04	5.20 ± 0.05	ND	ND	0.15 ± 0.01	0.55 ± 0.05
21.6	337		337.0923	337.0920	0.94	C16H17O8	cis-5-*p*-Cumaroylquinic acid	25.63 ± 0.07	27.63 ± 0.01	66.52 ± 0.77	70.37 ± 0.91	1.00 ± 0.08	2.20 ± 0.02	12.39 ± 0.10	11.90 ± 0.08
22.9	353	191	353.0873	353.0867	1.68	C16H17O9	3-Caffeoylquinic acid	2.38 ± 0.04	6.11 ± 0.08	0.19 ± 0.08	ND	0.22 ± 0.01	0.32 ± 0.05	0.41 ± 0.05	ND
23.5	353	191	353.0873	353.0869	1.08	C16H17O9	5-Caffeoylquinic acid	4.63 ± 0.08	4.59 ± 0.04	1.17 ± 0.06	13.00 ± 0.12	0.35 ± 0.02	1.11 ± 0.01	0.35 ± 0.01	5.37 ± 0.03
26.8	367		367.1029	367.1027	0.60	C17H19O9	Feruloylquinic acid	2.80 ± 0.01	3.43 ± 0.06	0.34 ± 0.06	0.40 ± 0.08	ND	ND	0.00	0.04 ± 0.08
28.9	337		—	337.0923	—	—	Feruoyl derivative	1.03 ± 0.01	6.32 ± 0.12	0.41 ± 0.04	0.80 ± 0.06	0.30 ± 0.06	ND	0.22 ± 0.01	0.28 ± 0.02
Small phenolics and PACs															
10.4	315		315.0716	315.0716	0.00	C13H15O9	Protocatechuic acid glucoside	4.79 ± 0.05	2.41 ± 0.03	10.32 ± 0.06	15.32 ± 0.06	1.12 ± 0.02	0.59 ± 0.08	9.94 ± 0.04	5.10 ± 0.04
25.5	577	451,425,407	577.1346	577.1349	−0.55	C30H25O12	PAC B dimer	38.12 ± 0.08	37.09 ± 0.05	25.77 ± 0.06	34.03 ± 0.17	103.16 ± 0.08	59.65 ± 0.11	14.65 ± 0.14	1.80 ± 0.02
26.8	865	739,576,289	865.1989	865.1993	−0.49	C45H37O18	PAC trimer	2.37 ± 0.05	29.51 ± 0.06	2.55 ± 0.01	0.72 ± 0.05	20.89 ± 0.07	6.51 ± 0.05	1.68 ± 0.05	ND
27.8	289		289.0712	289.0711	0.37	C15H13O6	Epicatechin^§^	51.19 ± 0.05	14.64 ± 0.05	9.39 ± 0.08	51.70 ± 0.16	113.38 ± 0.15	48.59 ± 0.09	30.68 ± 0.14	2.08 ± 0.05
28.9	577	451,425,407	577.1346	577.1349	−0.55	C30H25O12	PAC dimer	5.16 ± 0.04	22.13 ± 0.04	0.68 ± 0.07	3.44 ± 0.04	6.94 ± 0.05	4.39 ± 0.05	0.11 ± 0.02	ND
30.8	1153	576,289	1153.2614	1153.2628	−1.29	C60H49O24	PAC tetramer	2.50 ± 0.00	4.58 ± 0.02	9.10 ± 0.05	2.52 ± 0.05	84.68 ± 0.15	32.10 ± 0.12	0.32 ± 0.00	ND
31.4	1153	576,289	1153.2614	1153.2626	−1.10	C60H49O24	PAC tetramer	13.77 ± 0.05	1.16 ± 0.08	16.43 ± 0.05	4.95 ± 0.01	ND	3.29 ± 0.05	0.36 ± 0.09	ND
31.7	1153	576,289	1153.2614	1153.2628	−1.29	C60H49O24	PAC tetramer	26.50 ± 0.05	14.18 ± 0.05	6.34 ± 0.08	26.21 ± 0.14	84.68 ± 0.14	8.99 ± 0.10	8.69 ± 0.10	ND
32.0	865	739,576,289	865.1980	865.1993	−1.59	C45H37O18	PAC trimer	17.39 ± 0.05	54.47 ± 0.23	55.51 ± 0.21	25.85 ± 0.17	74.97 ± 0.18	20.02 ± 0.10	16.78 ± 0.10	13.03 ± 0.05
34.3	1153	576,289	1153.2614	1153.2625	−1.01	C60H49O24	PAC tetramer	6.84 ± 0.05	7.12 ± 0.08	15.93 ± 0.05	15.93 ± 0.05	8.08 ± 0.07	5.41 ± 0.03	3.79 ± 0.03	ND
34.5	865	739,576,289	865.1980	865.1990	−1.23	C45H37O18	PAC trimer	10.76 ± 0.01	1.74 ± 0.01	5.74 ± 0.00	1.06 ± 0.09	8.99 ± 0.02	23.81 ± 0.05	16.67 ± 0.05	ND
35	1441		1441.3248	1441.3247	0.07	C75H61O30	PAC pentamer	3.52 ± 0.06	5.01 ± 0.03	5.89 ± 0.09	7.42 ± 0.01	13.58 ± 0.01	13.54 ± 0.03	9.48 ± 0.05	ND
35.9	720***	644,577,407	—	—	—	—	PAC pentamer	ND	ND	ND	ND	6.28 ± 0.04	13.64 ± 0.15	9.55 ± 0.05	ND
36.9	1153	576,289	1153.2614	1153.2624	−1.01	C60H49O24	PAC tetramer	ND	ND	ND	ND	1.75 ± 0.05	2.34 ± 0.01	1.64 ± 0.01	ND
37	865	739,576,289	865.1980	865.1991	−1.23	C45H37O18	PAC trimer	ND	ND	ND	ND	2.06 ± 0.02	1.28 ± 0.05	0.90 ± 0.05	ND
38	865	739,576,289	865.1980	865.1991	−1.23	C45H37O18	PAC trimer	ND	ND	ND	ND	26.58 ± 0.16	12.14 ± 0.16	8.50 ± 0.18	ND
41	865	739,576,289	865.1980	865.1990	−1.23	C45H37O18	PAC trimer	7.08 ± 0.19	0.93 ± 0.11	10.25 ± 0.08	0.97 ± 0.15	5.17 ± 0.18	3.27 ± 0.15	ND	ND
41.9	1730		—	—	—	—	PAC derivative	4.17 ± 0.15	0.24 ± 0.05	3.18 ± 0.05	0.96 ± 0.07	1.80 ± 0.09	0.47 ± 0.02	6.94 ± 0.05	ND
Flavonoids and napthodiantrone derivatives															
34.3	479	316,287,271 243 179 151	479.0826	479.0823	0.67	C21H19O13	Myricetin hexoside	ND	ND	1.39 ± 0.01	1.75 ± 0.01	ND	ND	0.74 ± 0.02	1.86 ± 0.05
35.3	609		609.1456	609.1452	0.70	C27H29O16	Rutin^§^	70.24 ± 0.09	79.30 ± 0.23	ND	18.65 ± 0.02	ND	ND	0.23 ± 0.05	0.22 ± 0.04
35.3	463	301	463.0877	463.087	1.60	C21H19O12	Quercetin-3-galactoside (hyperoside)^§^	54.35 ± 0.12	13.15 ± 0.09	123.49 ± 0.33	46.27 ± 0.15	4.27 ± 0.04	4.06 ± 0.00	29.50 ± 0.16	6.60 ± 0.05
35.6	463	301	463.0877	463.0874	0.69	C21H19O12	Quercetin-3-glucoside^§^	15.87 ± 0.01	13.56 ± 0.05	26.34 ± 0.05	15.08 ± 0.03	5.46 ± 0.09	5.01 ± 0.06	8.50 ± 0.09	2.97 ± 0.09
36.8	433	300,271,255 179 151	433.0771	433.0769	0.49	C20H17O11	Quercetin 7-O-pentoside	ND	ND	43.10 ± 0.09	20.27 ± 0.10	ND	ND	12.32 ± 0.10	1.62 ± 0.09
37.1	433	301,271,179 151	433.0771	433.0766	1.22	C20H17O11	Quercetin 3-O-pentoside	ND	ND	38.65 ± 0.17	19.32 ± 0.12	ND	ND	12.92 ± 0.09	2.10 ± 0.01
37.6	447	301	447.0927	447.0928	-0.24	C21H19O11	Quercetin-3-rhamnoside	56.59 ± 0.15	56.72 ± 0.09	ND	0.34 ± 0.02	8.20 ± 0.05	3.57 ± 0.05	1.14 ± 0.01	0.66 ± 0.01
41	461		—	—	—	—	Flavonoid derivative	10.65 ± 0.00	11.61 ± 0.04	ND	ND	ND	ND	2.92 ± 0.06	2.21 ± 0.01
41.1	451	341,323,297 217 177	451.1029	451.1025	0.94	C24H19O9	Cinchonain-Ib	ND	ND	0.30 ± 0.09	1.48 ± 0.00	ND	ND	1.29 ± 0.00	1.00 ± 0.01
41.3	301		301.0348	301.0343	1.76	C15H9O7	Quercetin^§^	4.75 ± 0.10	1.95 ± 0.03	5.24 ± 0.06	0.80 ± 0.01	ND	ND	0.55 ± 0.03	0.06 ± 0.01
44.3	537	443,385	537.0822	537.0824	−0.39	C30H17O10	Biapigenin	6.72 ± 0.00	ND	ND	ND	ND	ND	ND	ND
44.5	537		537.0822	537.0826	−0.79	C30H17O10	Amentoflavone	0.73 ± 0.03	0.72 ± 0.03	3.43 ± 0.08	ND	ND	ND	ND	ND
59	503		503.0767	503.0766	−0.00	C30H15O8	Hypericin	1.56 ± 0.01	1.58 ± 0.03	1.49 ± 0.06	1.57 ± 0.07	1.66 ± 0.03	1.60 ± 0.01	1.18 ± 0.01	1.47 ± 0.00

HT-AP-MeOH: *H. triquetrifolium* aerial parts, methanol extract; HT-AP-Water: *H. triquetrifolium* aerial parts, water extract; HT-R-MeOH: *H. triquetrifolium* root, methanol extract; HT-R-Water: *H. triquetrifolium* root, water extract; HN-AP-MeOH: *H. neurocalycinum* aerial parts, methanol extract; HN-AP-Water: *H. neurocalycinum* aerial parts, water extract; HN-R-MeOH: *H. neurocalycinum* root, methanol extract; HN-R-Water: *H. neurocalycinum* root, water extract; ND: not detected. *: fragments in bold indicate the source of the MS3 fragments, reported in brackets; **: values obtained from LC-QTOF analysis; ***: ion detected as [M-2H]^2-^; ^§^: identification was confirmed by co-injection with reference standard.

#### Flavonoid Glycosides and Catechin, Procyanidin and Napthodiantrone Derivatives

*H. triquetifolium* and *H. neurocalycinum* extracts revealed the presence of epicatechin and procyanidin derivatives (PACs) up to pentamers. Protocatechuic acid glucoside was present in all the samples. Although, to the best of our knowledge, previous studies reporting procyanidins in *H. triquetifolium* and *H. neurocalycinum* have not been published, catechin and several oligomeric procyanidins (such as A2, B2, and B3) have been identified and isolated from *H. perforatum* (Ploss et al., 2001; Hellenbrand et al., 2015) and *H. hircinum* subsp. *Majus* (Tocci et al., 2018).

Among the flavonoid constituents of *H. triquetifolium* and *H. neurocalycinum* extracts, quercetin derivatives, rutin, quercetin-3-O-glucoside and hyperoside were detected in all the samples. Hypericin was also revealed in all the analysed extracts (Table 2).

#### Qualitative and Quantitative Differences in the Composition of the Tested Extracts

**FIGURE 4:**
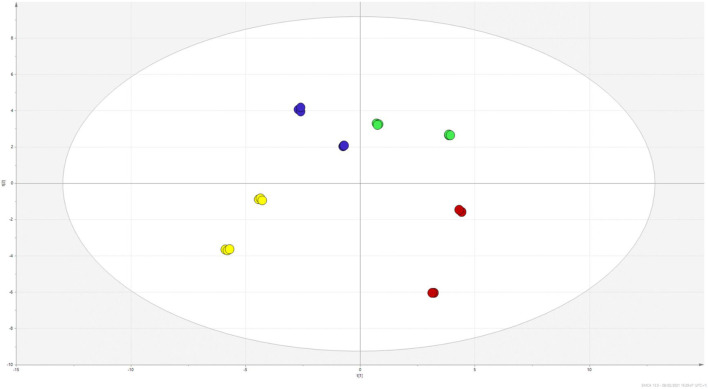
PCA scatter plot obtained from the quantitative data of *H. triquetifolium* aerial and roots extracts (red and yellow dots, respectively) and *H. neurocalicinum* aerial and roots extracts (green and blue dots, respectively).

Quantitative results are reported in Tables 1 and 2, summarising the amount of each identified metabolite. To observe differences between the two *Hypericum* species, the data matrix was initially elaborated using PCA. Results, reported the scatter plot in Figure 4, show a net clusterization: *H. neurocalycinum* samples are located in the upper part of the plot (green dots), while those of *H. triquetifolium* are grouped in the lower part (blue dots). The same plot shows also the clusterization of *Hypericum* samples based on the plant part extracted, i.e., roots and aerial parts populate respectively − x and + x parts of the plot. Differences between the two species could be ascribed to specific compounds, i.e. myricetin hexoside, quercetin-7-O-pentoside, quercetin-3-O-pentoside, cinchonain-Ib, 7-epiclusianone, hyperfirin, and hyperforin for *H. neurocalycinum*, and PAC trimer, PAC dimer and apigenin-7-O-glyucoside for *H. triquetifolium* (SupplementaryFigure S2).

**FIGURE 5:**
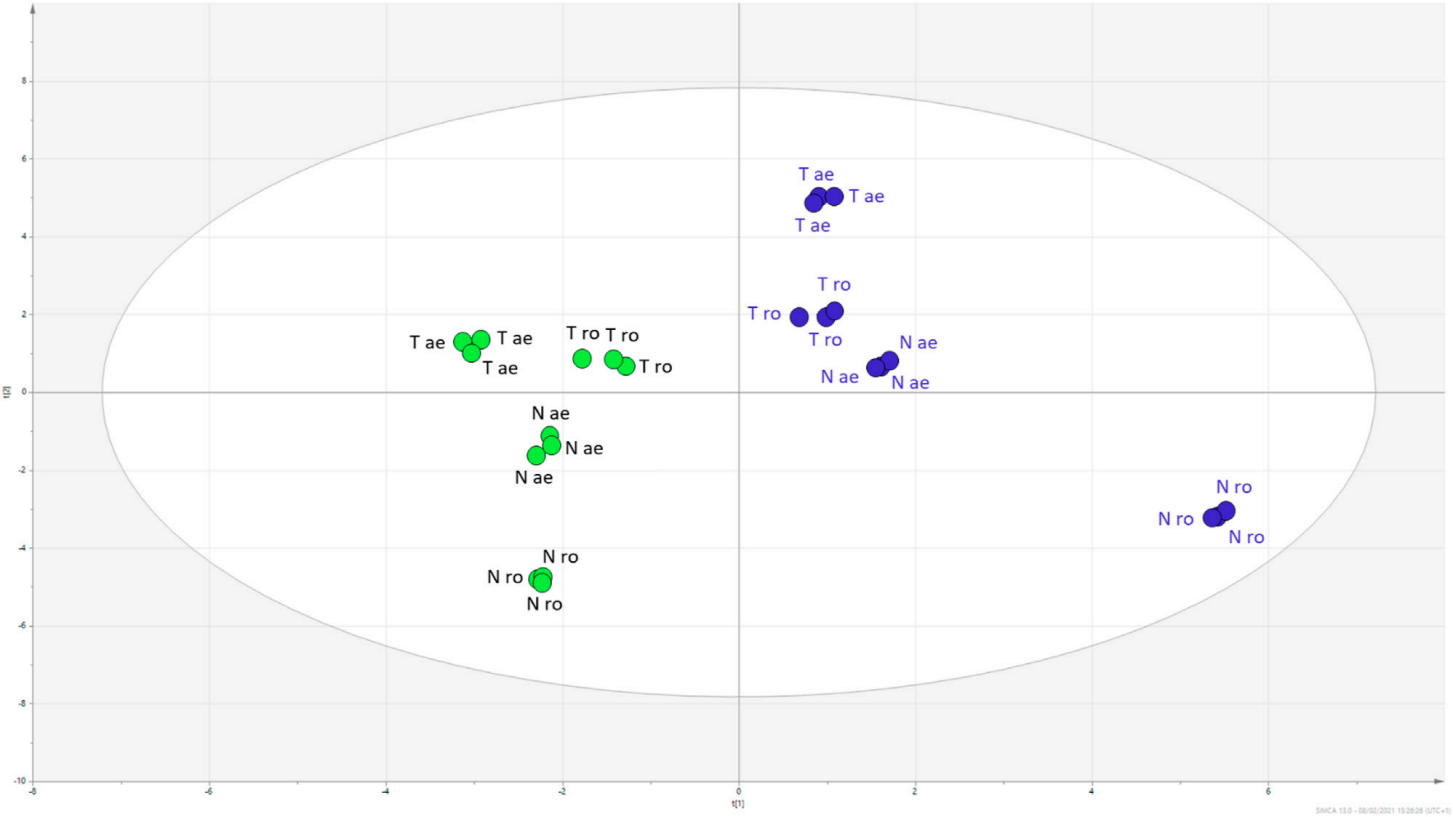
PLS-DA scatter plot of *H. triquetifolium* (T) and *H. neurocalicinum* (N) aerial (ae) and roots (ro) extracts obtained with either methanol (green dots) or water (blue dots).

The PCA indicated that limited variations occurred to the samples extracted with either methanol or water. To empathise the differences in composition related to the two extraction solvents, a supervised PLS-DA was performed, and the obtained plot is reported in Figure 5. The model was validated using the permutation test (1,000 random permutations), and the goodness of fit as well as the predictability of the model were expressed by R2X (cum) = 0.757 and R2Y (cum) = 0.997, and Q2 (cum) = 0.68, respectively. Overall, the parameters indicated the robustness of the model. Considering the VIP coefficients (>1) and the significance level calculated comparing methanol *vs.* water samples, we summarised the findings supporting the compounds related to different extraction solvents for the two considered plant parts (aerial parts and roots) in Supplementary Table S4. Furthermore, data are grouped in the Table showing the sum of mg/g of total hyroxycinnamic, small phenolics and PACs, total flavonoids, and total phloroglucinols. Considering the roots of *H. triquetifolium,* methanol extract reached more than 50% (w/w) of small phenolics and PACs, while water extract reached only 27% (w/w). A similar behaviour was observed for *H. neurocalycinum* roots, where small phenolics and PACs were extracted reaching 14% in methanol and 2.2% in water. Aerial parts of *Hypericum* species were rich in hydroxycinnamic acids, accounting for 100–185 mg/g, while 6–25 mg/g were measured from the roots. Flavonoids were, as expected, in large amount mainly in the aerial parts. Going further in detail on the different constituents that have been quantified, we observed some variation. Phloroglucinols presented a different behaviour: geranyl phlorisobutyrophenon, garsubellin E, and hyperpolyphyllirin, for example, were better extracted in methanol, while hyperfirin and 7-epiclusianone were better extracted in water. This finding might be related to the different solubility of each derivative in the two solvents as well as to the different composition of the tissues that were extracted, i.e., aerial parts and roots. Considering the phenolic constituents, a good extraction could be obtained with both the solvents. Some of the most lipophilic flavonoids as quercetin aglycone were better extracted with organic solvent, while for most of the hydroxycinnamic derivatives water extraction appeared to be more effective than methanol.

### Antioxidant Properties of H. triquetrifolium and H. neurocalycinum Extracts

Multiple assays were performed to assess the antioxidant capacity of the extracts and the results are given in Table 3. The total antioxidant capacity of the extracts was measured by recording the absorbance of green phosphate/Mo(V) complex formed in acidic condition (Boroja et al., 2018). It can be noted from Table 3 that the methanol and water extracts of *H. triquetrifolium* aerial parts (3.28–3.61 mmol TE/g for methanol and water extracts, respectively) and roots (3.36–3.02 mmol TE/g for methanol and water extracts, respectively) exerted a higher antioxidant activity compared to *H. neurocalycinum* extracts (1.35–2.59 mmol TE/g). Metal chelation is a crucial antioxidant defence mechanism. In fact, metal chelators can sequester metal ions by forming cyclic coordination complexes (Howard and Wilson, 2003). The participation of metal ions such as iron species in Fenton reaction generate oxidizing species which contribute to oxidative stress and cause oxidative damages to biomolecules (Zhao, 2019). In the present study, *H. neurocalycinum* extracts demonstrated the highest metal chelation properties (Table 3). Among the *H. neurocalycinum* extracts, the water extract of *H. neurocalycinum* roots exhibited the highest metal chelating activity (30.00 mg EDTAE/g), while the methanol extract of *H. triquetrifolium* aerial parts exhibited the lowest activity.

**TABLE 3:** *In vitro* antioxidant properties of *Hypericum triquetrifolium* and *Hypericum neurocalycinum* extracts.

*Hypericum* species	Parts/solvents	DPPH (mg TE/g)	ABTS (mg TE/g)	CUPRAC (mg TE/g)	FRAP (mg TE/g)	Phosphomolybdenum (mmol TE/g)	Metal chelating ability (mg EDTAE/g)
*H. neurocalycinum*	Aerial parts-MeOH	251.90 ± 5.35^d^	473.77 ± 3.04^d^	591.69 ± 7.16^c^	305.34 ± 0.75^d^	2.59 ± 0.11^d^	21.87 ± 0.64^d^
	Aerial parts-Water	288.01 ± 3.40^c^	400.89 ± 5.40^e^	484.12 ± 1.39^d^	285.40 ± 1.11^f^	2.32 ± 0.05^d^	26.04 ± 0.17^b^
	Roots-MeOH	121.10 ± 1.58^e^	335.69 ± 14.35^f^	318.81 ± 3.82^e^	157.60 ± 1.95^g^	1.86 ± 0.06^e^	22.13 ± 0.26^cd^
	Roots-water	121.83 ± 1.64^e^	176.28 ± 0.96^g^	223.42 ± 1.29^f^	118.51 ± 1.44^h^	1.35 ± 0.05^f^	30.00 ± 0.19^a^
*H. triquetrifolium*	Aerial parts-MeOH	325.76 ± 13.44^b^	517.19 ± 5.43^c^	602.27 ± 14.38^bc^	297.75 ± 1.39^e^	3.28 ± 0.18^bc^	12.61 ± 0.71^f^
	Aerial parts-Water	400.42 ± 10.03^a^	628.81 ± 22.46^a^	694.90 ± 4.98^a^	434.76 ± 0.34^a^	3.61 ± 0.11^a^	17.20 ± 0.29^e^
	Roots-MeOH	343.17 ± 11.60^b^	617.53 ± 27.45^a^	610.04 ± 4.58^b^	327.29 ± 5.47^c^	3.36 ± 0.17^ab^	23.24 ± 0.54^c^
	Roots-water	407.35 ± 8.76^a^	556.53 ± 2.00^b^	608.08 ± 1.59^bc^	337.30 ± 1.44^b^	3.02 ± 0.11^c^	26.88 ± 0.07^b^

*Values expressed are means ± S.D. of three parallel measurements. TE, Trolox equivalent; EDTAE: EDTA equivalent. Different superscripts ^(a-h)^ in the same column indicate significant differences in the extracts (*p* < 0.05 from one-way ANOVA followed by Post Hoc Tukey test is considered significant; the superscript “^a^” indicates the highest activity).

The reducing potential of the studied *Hypericum* species was evaluated using the standard FRAP and CUPRAC methods. Interestingly, the water extract of *H. triquetrifolium* aerial parts showed highest reducing potential (694.90 and 434.76 mg TE/g). Earlier studies have reported the correlation between phenolic content and reducing properties (Upadhyay et al., 2014; Boroja et al., 2018). Likewise, this observation has been recorded for radical scavenging studies. In the present study, it was observed that the water extract of *H. triquetrifolium* aerial parts showed highest activity against ABTS radical, while the water extract of *H. triquetrifolium* root was more active against the DPPH radical. For the assessment of antioxidant activity, it has been advocated that the ABTS assay is more sensitive due to its faster reaction kinetics and its higher response to antioxidants (Lee et al., 2015). Previous studies have reported the antioxidant activity of the methanol extract of the aerial parts of *H. neurocalycinum* and *H. triquetrifolium*, but a paucity of scientific information regarding the antioxidant activity of the water extracts of these two *Hypericum* species was noted (Conforti et al., 2002; Ozkan et al., 2013; Eroglu Ozkan et al., 2018).

### Evaluation of Enzyme Inhibitory Activity

Numerous investigations have attempted to harness the enzyme inhibitory potential of natural compounds for the development of novel drug candidates. Endeavors to discover and develop lead compounds are focused on the optimization of candidates that can target specific enzymes, since enzymes characterize high level of disease association, i.e., target validation and druggability, and target tractability (Copeland et al., 2007). Here, the possible inhibitory action of methanol and water extracts of *H. neurocalycinum* and *H. triquetrifolium* aerial parts and roots was determined. As shown in Table 4, extracts of the studied *Hypericum* species exhibited poor inhibition on α-amylase and α-glucosidase, of which the latter has been claimed to be an interesting target for the management of diabetes type II due to less adverse effects compared to traditional treatments. It is worth mentioning that only methanol extracts exhibited some activity, while water extracts were not active against α-glucosidase. The aerial parts of another species, namely *H. perforatum* subsp. *perforatum,* have shown to exhibit inhibitory activity against both α-amylase and α-glucosidase (Kladar et al., 2017). The authors reported that the inhibitory effects were 51.10% (for α-amylase at 400 μg/ml) and 41.33% (for α-glucosidase at 400 μg/ml). Taken together, the members of the *Hypericum* genus could be considered as valuable sources of α-amylase and α-glucosidase inhibitors.

**TABLE 4:** *In vitro* enzyme inhibitory effects of *Hypericum triquetrifolium* and *Hypericum neurocalycinum* extracts.

*Hypericum* species	Parts/solvents	AChE inhibition (mg GALAE/g)	BChE inhibition (mg GALAE/g)	Tyrosinase inhibition (mg KAE/g)	Amylase inhibition (mmol ACAE/g)	Glucosidase inhibition (mmol ACAE/g)
*H. neurocalycinum*	Aerial parts-MeOH	2.13 ± 0.29^ab^	3.05 ± 0.07^b^	67.45 ± 0.46^b^	0.90 ± 0.03^a^	0.97 ± 0.02^a^
	Aerial parts-Water	0.73 ± 0.09^d^	0.92 ± 0.05^c^	13.96 ± 2.02^e^	0.21 ± 0.01^d^	na
	Roots-MeOH	1.60 ± 0.16^c^	3.70 ± 0.38^ab^	65.64 ± 0.63^b^	0.80 ± 0.03^b^	0.91 ± 0.03^b^
	Roots-water	0.63 ± 0.08^d^	1.10 ± 0.25^c^	na	0.17 ± 0.01^d^	na
*H. triquetrifolium*	Aerial parts-MeOH	2.05 ± 0.04^b^	3.59 ± 0.50^b^	66.74 ± 0.20^b^	0.80 ± 0.01^b^	0.96 ± 0.02^ab^
	Aerial parts-Water	1.55 ± 0.07^c^	0.53 ± 0.03^c^	40.35 ± 0.27^c^	0.91 ± 0.01^a^	na
	Roots-MeOH	2.48 ± 0.02^a^	4.38 ± 0.29^a^	69.93 ± 0.14^a^	0.81 ± 0.04^b^	0.96 ± 0.03^ab^
	Roots-water	1.41 ± 0.02^c^	0.62 ± 0.02^c^	33.89 ± 0.47^d^	0.34 ± 0.01^c^	na

* Values expressed are means ± S.D. of three parallel measurements. GALAE: Galatamine equivalent; KAE: Kojic acid equivalent; ACAE: acarbose equivalent; na: not active. Different superscripts ^(a-h)^ in the same column indicate significant differences in the extracts (*p* < 0.05 from one-way ANOVA followed by Post Hoc Tukey test is considered significant; the superscript “^a^” indicates the highest activity).

As shown in Table 4, the extracts of the studied *Hypericum* species exhibited inhibitory potential on AChE and BChE. Previously, it has been reported that the methanol extract of *H. neurocalycinum* (5 mg/ml) inhibited 72.24% of AChE activity (Eroglu Ozkan et al., 2018). Likewise, in the present study, the methanol extract of *H. neurocalycinum* aerial parts (2.13 mg GALAE/g) showed higher inhibitory activity against AChE compared to the other extracts studied (0.63–1.60 mg GALAE/g). However, the methanol extract of *H. triquetrifolium* roots (2.48 mg GALAE/g) showed even higher activity. Although the inhibition of AChE is considered to be a highly viable strategy for the symptomatic management of Alzheimer's disease, the role of BChE in late Alzheimer’s disease has been recognized. In the present study, the methanol extract of *H. triquetrifolium* roots (4.38 mg GALAE/g) was found to exhibit high inhibition against BChE (Mehta et al., 2012). In a previously published study, *H. humifusum* exhibited higher inhibition on AChE (4.57 mg GALAE/g), α-amylase (2.55 mmol ACE/g), and α-glucosidase (8 mmol ACE/g) (Béjaoui et al., 2017). When compared to our findings, *H. humifusum* exhibited similar AChE inhibitory effects of the tested *Hypericum* species, but its amylase and glucosidase inhibitory effects were higher than those of both *Hypercium* species.

Tyrosinase is a rate limiting enzyme responsible for the biosynthesis of melanin, which is considered as a key therapeutic strategy for the management of skin hyperpigmentation conditions (Zolghadri et al., 2019). In the present study, the methanol extracts of *H. neurocalycinum* and *H. triquetrifolium* aerial parts and roots exhibited high inhibition against tyrosinase. The methanol extract of *H. triquetrifolium* roots displayed the best tyrosinase inhibitory ability (69.93 mg KAE/g extract). On the contrary, the water extract of *H. neurocalycinum* was not active on tyrosinase.

### Data Mining

A PLS-DA model was developed to build an accurate species classification model based on biological data. PLS-DA is a useful supervised multivariate tool dealing with complex data: it minimizes background effects and provides an effective descriptive and predictive modelling of the data itself. It has been used in numerous scientific areas such as genomics, pharmaceutical science, lipidomics, proteomics, and many others (Zontov et al., 2020). Figure S3A shows the samples plot for function 1 *vs*. function 2: as illustrated, the samples of *H. neurocalycinum* are separated from *H. triquetrifolium* along the first function of the model. The k-fold cross-validation of the model is reported in Supplemental Figure S3B. It can be observed from the classification error rate (BER) and the maximum distance, the best performance of the model seemed to be achieved for function = 1. Additionally, the AUC value is 1, indicating that there is 100% chance that the first function of the model will be able to discriminate both studied species (Supplemental Figure S3B). Thus, the PLS-DA model can successfully distinguish between *H. neurocalycinum* and *H. triquetrifolium*.

The identification of differential bioactivities between both the species was carried out using the VIP values. Thus, taking into account the VIP index greater than 1, phosphomolybdenum, DPPH, ABTS, FRAP, and CUPRAC could be considered as the potential assays for distinguish the two species (Supplemental Figure S3C). This result entailed that the antioxidant properties played a crucial role for *H. neurocalycinum* and *H. triquetrifolium* discrimination, probably due to the presence/absence and/or great/lower quantity of compounds responsible for the observed antioxidant properties. Afterwards, through comprehensive comparison using Student’s *t*-test, superior ABTS and DPPH radical scavenging, CUPRAC and FRAP reducing ability and total antioxidant capacity were recorded for *H. triquetrifolium* samples (Figure S3D).

### Correlation Between Phytochemical Characterization and Bioactivities

**FIGURE 6:**
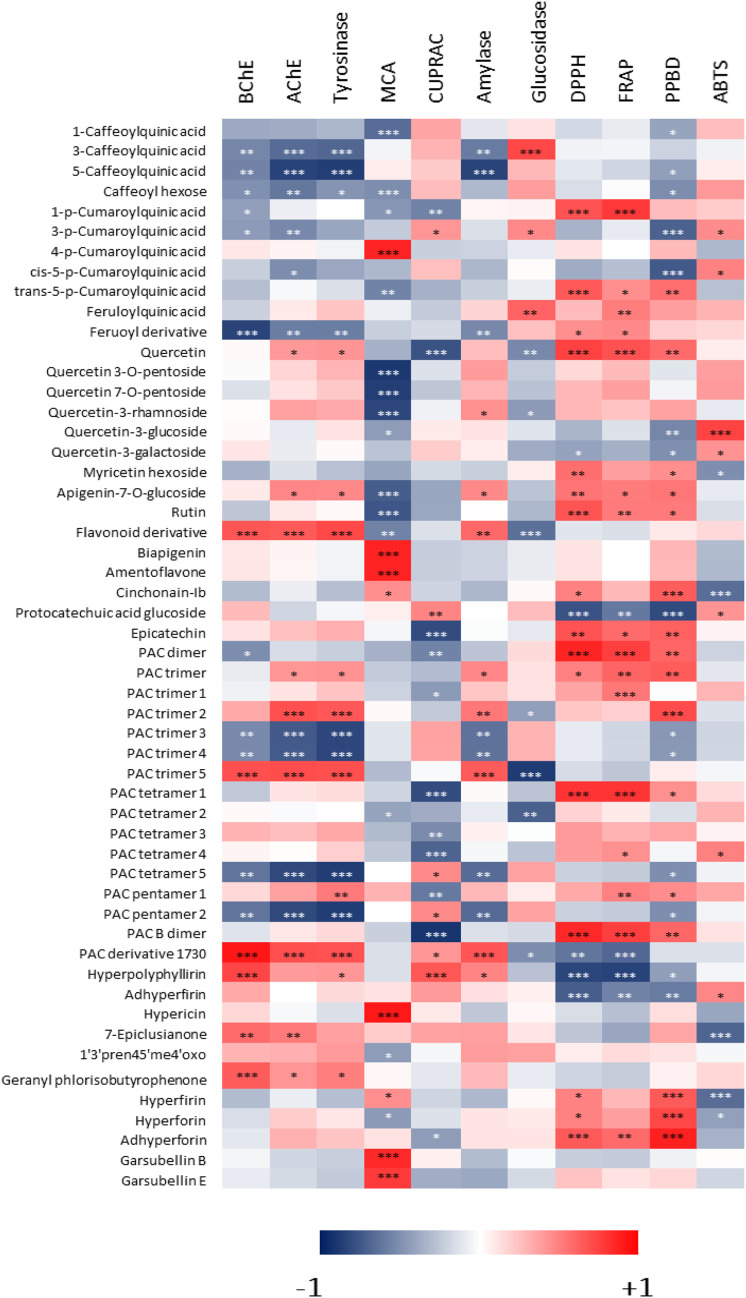
Heatmap showing the correlations among phenolic compounds identified in *Hypericum* methanol extracts and measured biological activities. *: *p*-value < 0.05; **: *p*-value < 0.01; ***: *p*-value < 0.001.

Correlation analysis was performed to determine if the compounds identified in *Hypericum* extracts could be specifically related to the measured biological activities of the different samples. The results, reported in the heatmap in Figure 6, show that the presence of specific constituents is positively correlated to the bioactivities exerted by the extracts, while other constituents are not significantly contributing to the same activities, thus yielding in a negative contribution. In a general way, this type of analysis could help to obtain a preliminary indication about the constituents that contribute the most to the observed bioactivities.

Considering BChE, AChE and tyrosinase, the heatmap in Figure 6 indicates that some specific constituents were related to the observed effects. For example, PAC trimer five and PAC derivative with *m/z* 1730 were positively correlated with these activities, while other PAC trimers appeared to be negatively correlated. This suggests that for these enzymatic inhibitory effects, PACs can be at least in part responsible, nevertheless changes in structure can lead to significant decrease or increase of the activity. Specific evaluation of purified compounds should be performed to assess the contribution of different derivatives. Furthermore, some hydroxycinnamic acid derivatives were negatively correlated with the inhibition of these enzymes. Data indicate that hypopolyphyllirine and geranyl phloroisobutyrophenone were positively correlated with BChE, suggesting a certain specificity of effect. Further data should be obtained testing the single compounds to better understand the role of phloroglucinols.

With respect to the chelation of metals, two different assays were performed, namely metal chelating activity and CUPRAC. In the first assay, the most significant positive correlations (*p* < 0.001) were observed for 4-coumaroylquinic acid, biapigenin, amentoflavone, hypericin, garsubellin B, and garsubellin E. On the other hand, several flavonoid glycosides were negatively correlated with this activity. For CUPRAC, a strong (*p* < 0.001) positive correlation was observed only with hyperpolyphyllirin. 5-caffeoylquinic acid was negatively correlated with amylase inhibition, while PAC trimer 5 and PAC derivative with *m/z* 1,730 were positively correlated with the effect on this enzyme. The effect was different on glucosidase, in fact 3-caffeoylquinic acid was positively correlated, while PAC trimer 5 was negatively correlated with its inhibition. 3-Caffeoylquinic acid and feruloylquinic acid were positively correlated with α-glucosidase activity, while compounds correlated with α-amylase were some PAC derivatives. The data indicate that mainly procyanidins and simple quinic acid esters with hydroxycinnamic derivatives may exert inhibitory activity on the two enzymes related to sugar metabolism.

## Conclusion

*H. neurocalycinum* and *H. triquetrifolium* from Turkey showed different phytochemical compositions, and using multivariate data analysis markers of each species were identified. Specifically, myricetin hexoside, quercetin-7-O-pentoside, quercetin-3-O-pentoside, cinchonain-Ib, 7-epiclusianone, hyperfirin, and hyperforin were assessed as markers for *H. neurocalycinum*, while PAC trimer, PAC dimer and apigenin-7-O-glyucoside were those for *H. triquetifolium*. Comparing methanol *vs.* water extractions, the former was in general more effective for the extraction of phloroglucinols, phenolic acids, PACs and flavonoids. Considering the biological assays, both *Hypericum* species showed significant antioxidant and metal chelating activities, as well as a significant inhibitory effect on tyrosinase. Moderate inhibitory activities were observed against AChE and BChE, while a weak effect on the inhibition of α-amylase and α-glucosidase was observed. In general, methanol extracts were more active than aqueous ones, and this could be explained by the higher phenolic content of the former. Finally, correlation analysis allowed to observe preliminary correlations between specific compounds among those identified and the assayed bioactivities. These results indicate that the integration of comprehensive phytochemical screening, bioactivity assays and multivariate analysis can afford a suitable and rapid approach for the identification of compounds with specific biological activities in complex natural extracts, and it could guide further isolation studies on the most promising compounds.

## Data Availability

The raw data supporting the conclusions of this article will be made available by the authors, without undue reservation.
