# Supplementary material for: Hypericum triquetrifolium and H. neurocalycinum as Sources of Antioxidants and Multi-Target Bioactive Compounds: A Comprehensive Characterization Combining In Vitro Bioassays and Integrated NMR and LC-MS Characterization by Using a Multivariate Approach
Source: Front Pharmacol. 2021 Mar 26;12:660735. doi: 10.3389/fphar.2021.660735 (PMC8033251; doi:10.3389/fphar.2021.660735)
Supplement: Supplementary file 1 [file datasheet1.docx]

**SUPPLEMENTARY MATERIAL**

**Table S1.** Total phenolic and flavonoid contents of *Hypericum neurocalycinum* and *H. triquetrifolium.*

| ***Hypericum* species** | **Parts-solvents** | **Total phenolic content (mg GAE/g)** | **Total flavonoid content (mg RE/g)** |
| --- | --- | --- | --- |
| *H. neurocalycinum* | Aerial parts-MeOH | 108.73±1.13 ^c^ | 125.71±0.70 ^a^ |
|  | Aerial parts-Water | 104.11±0.72 ^d^ | 71.70±0.22 ^d^ |
|  | Roots-MeOH | 79.23±0.51 ^e^ | 61.25±0.56 ^e^ |
|  | Roots-Water | 55.41±0.31 ^f^ | 12.82±0.39 ^g^ |
| *H. triquetrifolium* | Aerial parts-MeOH | 116.76±0.68 ^b^ | 122.85±0.60 ^b^ |
|  | Aerial parts-Water | 119.95±0.59 ^a^ | 89.63±0.96 ^c^ |
|  | Roots-MeOH | 120.01±0.51 ^a^ | 29.79±0.16 ^f^ |
|  | Roots-Water | 110.28±1.85 ^c^ | 10.41±0.20 ^h^ |
| * Values expressed are means ± S.D. of three parallel measurements. GAE: Gallic acid equivalent; RE: Rutin equivalent. Different superscripts ^(a-h)^ in the same column indicate significant differences in the extracts (*p*<0.05 from one-way ANOVA followed by Post Hoc Tukey test is considered significant; the superscript “^a^” indicates the highest activity). | | | |

**Table S2.** Main NMR assignments of compounds present in the methanol/water (50-50%) ATM fraction of *H. triquetifolium* aerial parts.

| **Compounds** | **Position** | **δ_H_** | **δ_C_ HSQC** | **δ_H_ COSY** | **δ_C_ HMBC** |
| --- | --- | --- | --- | --- | --- |
| **Phenolic constituents** |  |  |  |  |  |
| Caffeoyl and *p-*coumaroyl moiety | 7  8 | 7.67-7.60  6.38-6.30 | 148  147 | 6.38  7.67 | 168 116  168 116 |
| *p*-Coumaroyl moiety | 2-6 | 7.50 | 133 | 6.90 | 133 160 147 129 |
| Flavonol/catechin | 6  8 | 6.27  6.43 | 101  96 |  | 165 161 104 96  165 161 104 101 |
| 1,3,4-Trisubstitued aromatic ring | 2’  6’ | 7.36  7.60 | 118  125 | 7.60  7.36 6.70 | 144 146 125  144 146 118 |
| Protocatechuic acid | 2  5  6 | 7.86  6.90  7.61 | 119  115  121 | 7.61  7.61  6.90 | 121 147 144 115 |
| Quercetin derivatives (hyperoside) | 2’  6’  5’ | 7.72  7.65  6.89 | 116  123  118 | 7.65  7.72 6.89  7.65 | 158 147 146 124 |
| Glycosidic substituent Rhamnopyranosyl unit | 1”  2”  3”  4”  5”  6” | 5.37(d, J=1.5)  4.24 (dd, J=1.6; 3.0)  3.77 m  3.36 m  3.43 m  0.94 (d, J=6.53) | 104.9  73.5  79.6  73.4  75.0  19.1 | 4.24  3.77 5.37  4.24 3.36  3.77 3.43  3.36 0.94  3.43 | 137.8  75.0 73.4 |
| Glycosidic substituent  Rhamnopyranosyl unit (rutin) | 1”  2”  3”  4”  5”  6” | 4.54 (d, J=1.5)  3.65  3.31 m  3.56 m  3.46 m  1.14 (d, J=6.23) | 103.9  78.0  19.1 |  | 70 73.4  75.0 71.4 |
| Glycosidic substituent  Glucopyranosyl unit (rutin) | 1”  2”  3”  4”  5”  6” | 5.13 (d, J=8.0)  3.50  3.31 m  3.56 m  3.46 m  3.40 | 106.0  70.6 | 3.50 |  |
| Glycosidic substituent (hexosyl unit) | 1”  2’’  3’’  4’’  5’’  6’’ | 5.25 (d, J=7.7)  3.50 m  3.45 m  3.38 m  3.24 m  3.49-3.68 | 105.7  77.0  75  79  76  67 | 3.50 | 136.8 |
| Glycosidic substituent [galactopyranosyl unit (hyperoside)] | 1”  2”  3”  4”  5”  6” | 5.15 (d, J=7.7)  3.85  3.60  3.81  3.48  4.11 | 106.7  73  75.8  70  78.0  62.9 | 3.85 | 137.3 |
| Glycosidic substituent  (glucopyranosil unit) | 1” | 5.11 (d, J=8.0) | 106.0 | 3.50 | 137.1 |
|  |  |  |  |  |  |
| **Saccharides** |  |  |  |  |  |
| Saccharose | 1  5’ | 5.40 (d, J=4.0)  - | 95.1  106.7 | 3.46 3.74 3.83 | 106.7 76.0 |
| β-glucose | 1 | 4.50 (d, J=7.6) | 100.0 |  |  |
| α-glucose | 1 | 5.13 | 95.4 | 3.38 |  |
| Fructose | 4 | 4.0 | 77.0 |  |  |
|  |  |  |  |  |  |
| **Aliphatic signals** |  |  |  |  |  |
| Quinic acid derivatives | CH_2_ | 1.92-2.14 | 43.5 | 3.98 | 75.0 |
|  | CH_2_ | 2.15 | 38.5 | 4.16 |  |
|  | CH_2_ | 2.06 | 39.0 | 4.01 |  |

**Table S3.** Main NMR assignments of compounds present in the methanol ATM fraction of *H. triquetifolium* aerial parts.

| **Phloroglucinol constituents** | **Position** | **δ_H_** | **δ_C_ HSQC** | **δ_H_ COSY** | **δ_C_ HMBC** |
| --- | --- | --- | --- | --- | --- |
| CH sp^2^ prenyl moieties | 17  22  27 | 5.21  5.14  5.05 | 126.8  126.8  127.2 | 1.65  1.65  1.65 | 24.0 16.8  24.0 16.8  24.0 16.8 |
| CH_3_ prenyl moieties | 19,25  20, 24, 30  29 | 1.72  1.65  1.72 | 18.8  27.5  19.6 |  | 132.9 122.4 16.9  135.6 115.6 24.1  133.0 24.3 |
| CH_3_ keto-isobutyl moieties | 12  13 | 1.08  1.05 | 21.1  22.3 |  | 214.0 31.6 |

**Table S4.** Variations of compounds identified as discriminant for water and methanol extractions in aerial parts and root samples. Variables with VIP >1 were selected.

| **Compounds discriminant for aerial parts extracts** | **MeOH** | **Water** | **p-value** |
| --- | --- | --- | --- |
| 1-Caffeoylquinic acid | 26.96 ± 10.19 | 38.23 ± 25.38 | 0.34 |
| trans-5-p-Cumaroylquinic acid | 15.27 ± 16.2 | 9.9 ± 9.22 | 0.50 |
| 4-p-Cumaroylquinic acid | 1.68 ± 0.26 | 0.04 ± 0.05 | <0.001* |
| cis-5-p-Cumaroylquinic acid | 45.78 ± 22.37 | 48.82 ± 23.68 | 0.82 |
| PAC tetramer 2 | 15.16 ± 1.45 | 3.05 ± 2.07 | <0.001* |
| Rutin | 35.21 ± 38.57 | 48.94 ± 33.33 | 0.52 |
| Quercetin-3-galactoside (hyperoside) | 89.26 ± 38.22 | 29.65 ± 18.13 | 0.006* |
| Quercetin-3-glucoside | 21.12 ± 5.72 | 14.35 ± 0.87 | 0.017* |
| Quercetin | 4.99 ± 0.3 | 1.37 ± 0.62 | <0.001* |
| Amentoflavone | 2.09 ± 1.48 | 0.35 ± 0.39 | 0.02* |
| Adhyperfirin | 1.68 ± 0.07 | 1.3 ± 0.68 | 0.2 |
|  |  |  |  |
| **Compounds discriminant for root extracts** | **MeOH** | **Water** | **p-value** |
| PAC pentamer 1 | 11.51 ± 2.23 | 6.77 ± 7.41 | 0.16 |
| PAC pentamer 2 | 7.93 ± 1.78 | 6.8 ± 7.45 | 0.73 |
| PAC tetramer 5 | 1.71 ± 0.06 | 1.18 ± 1.29 | 0.34 |
| PAC trimer 3 | 1.47 ± 0.63 | 0.64 ± 0.71 | 0.05* |
| PAC trimer 4 | 17.56 ± 9.89 | 6.08 ± 6.66 | 0.04* |
| *Statistically significant variations between MeOH and water extracts | | | |

**Figure S1.** Chemical structures of the phenolic compounds assigned by NMR.


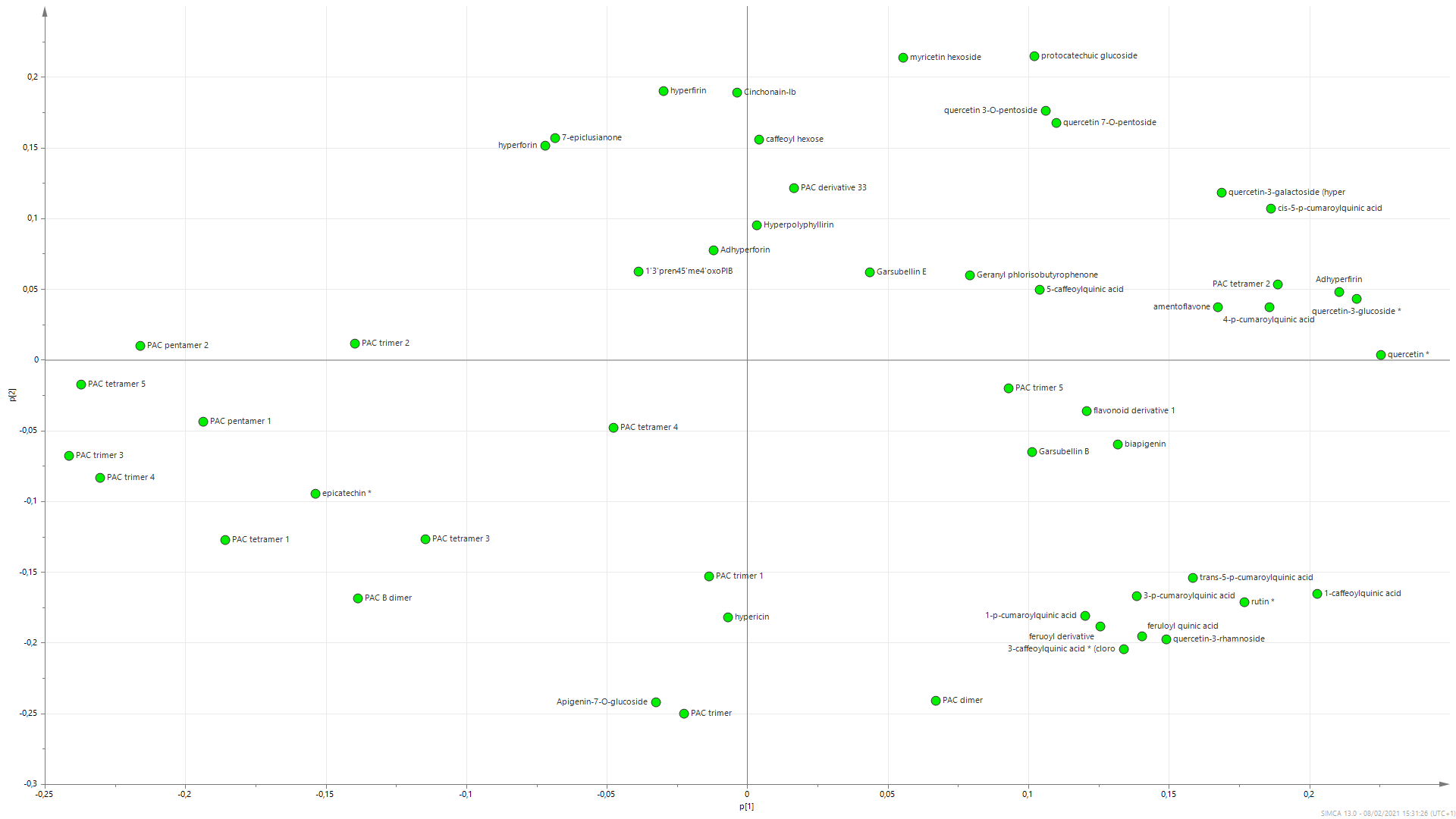


**Figure S2.** PCA loading scatter plot obtained from the quantitative data of *H. triquetifolium* and *H. neurocalicinum* aerial and roots extracts.


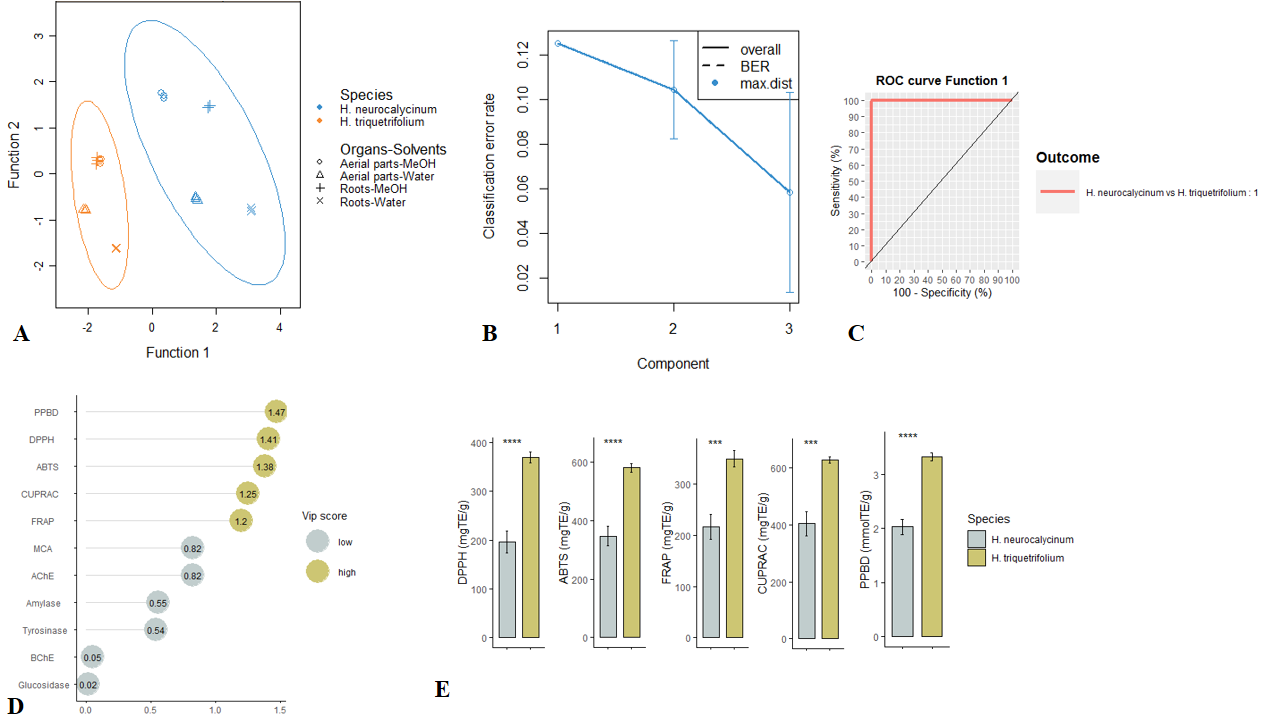


**Figure S3.** Partial least squares discriminant analysis (PLS-DA) on the bioactivities of *H. neurocalycinum* and *H. triquetrifolium* samples. A: score plot separating the samples according to the studied species. B and C: goodness of the PLS-DA model assessed using k-fold cross-validation and Area Under the Curve average using one-vs-all comparisons. D: VIP plot showing the most discriminant bioactivities. E: Comparison between the species considering the most discriminant bioactivities.
